# How Changes in Anti-SD Sequences Would Affect SD Sequences in *Escherichia coli* and *Bacillus subtilis*

**DOI:** 10.1534/g3.117.039305

**Published:** 2017-03-31

**Authors:** Akram Abolbaghaei, Jordan R. Silke, Xuhua Xia

**Affiliations:** * Department of Biology, University of Ottawa, Ontario K1N 6N5, Canada; † Ottawa Institute of Systems Biology, Ontario K1H 8M5, Canada

**Keywords:** ssu rRNA, *Escherichia coli*, *Bacillus subtilis*, Shine-Dalgarno, anti-SD-sequence, translation efficiency

## Abstract

The 3′ end of the small ribosomal RNAs (ssu rRNA) in bacteria is directly involved in the selection and binding of mRNA transcripts during translation initiation via well-documented interactions between a Shine-Dalgarno (SD) sequence located upstream of the initiation codon and an anti-SD (aSD) sequence at the 3′ end of the ssu rRNA. Consequently, the 3′ end of ssu rRNA (3′TAIL) is strongly conserved among bacterial species because a change in the region may impact the translation of many protein-coding genes. *Escherichia coli* and *Bacillus subtilis* differ in their 3′ ends of ssu rRNA, being GAUC**ACCUCCUUA**3′ in *E. coli* and GAUC**ACCUCCUU**UCU3′ or GAUC**ACCUCCUU**UCUA3′ in *B. subtilis*. Such differences in 3′TAIL lead to species-specific SDs (designated SD_Ec_ for *E. coli* and SD_Bs_ for *B. subtilis*) that can form strong and well-positioned SD/aSD pairing in one species but not in the other. Selection mediated by the species-specific 3′TAIL is expected to favor SD_Bs_ against SD_Ec_ in *B. subtilis*, but favor SD_Ec_ against SD_Bs_ in *E. coli*. Among well-positioned SDs, SD_Ec_ is used more in *E. coli* than in *B. subtilis*, and SD_Bs_ more in *B. subtilis* than in *E. coli*. Highly expressed genes and genes of high translation efficiency tend to have longer SDs than lowly expressed genes and genes with low translation efficiency in both species, but more so in *B. subtilis* than in *E. coli*. Both species overuse SDs matching the bolded part of the 3′TAIL shown above. The 3′TAIL difference contributes to the host specificity of phages.

Many studies suggest that initiation is the principle bottleneck of the translation process in bacteria (Liljenstrom and von Heijne 1987; Bulmer 1991; Xia 2007a; Xia *et al.* 2007; Kudla *et al.* 2009; Tuller *et al.* 2010; Prabhakaran *et al.* 2015). Successful initiation requires that the ribosome is able to bind to the mRNA template in such a manner that the start codon correctly lines up at the ribosomal P site (Farwell *et al.* 1992; Komarova *et al.* 2002; Duval *et al.* 2013). This translation initiation process in most bacterial species is facilitated by (1) ribosomal protein S1 (RPS1) acting as an RNA chaperone that unfolds secondary structural elements that may otherwise embed the start codon and obscure the start signal (Vellanoweth and Rabinowitz 1992; Duval *et al.* 2013; Prabhakaran *et al.* 2015), and (2) the Shine-Dalgarno (SD) sequence located upstream of the start codon (Shine and Dalgarno 1974, 1975; Steitz and Jakes 1975; Dunn *et al.* 1978; Taniguchi and Weissmann 1978; Eckhardt and Luhrmann 1979; Luhrmann *et al.* 1981) that base-pairs with anti-SD (aSD) located at the free 3′ end of the small ribosomal rRNA (ssu rRNA, whose 3′ end will hereafter be referred to as 3′TAIL). A well-positioned SD/aSD pairing and reduced secondary structure in sequences flanking the start codon and SD are the hallmarks of highly expressed genes in *Escherichia coli* and *Staphylococcus aureus*, as well as their phages (Prabhakaran *et al.* 2015).

The SD/aSD pairing offers a simple and elegant solution to start codon recognition in bacteria and their phages (Hui and de Boer 1987; Vimberg *et al.* 2007; Prabhakaran *et al.* 2015). Because many protein-coding genes depend on aSD motifs located at 3′TAIL for translation, strong sequence conservation is observed in the 3′TAIL among diverse bacterial species (Woese 1987; Orso *et al.* 1994; Clarridge 2004; Chakravorty *et al.* 2007). Conversely, a change in 3′TAIL is expected to result in fundamental changes in SD usage in protein-coding genes.

*E. coli*, as a representative of the gram-negative bacteria, and *Bacillus subtilis*, as a representative of gram-positive bacteria, differ in their 3′TAIL in only a minor detail, with the former ending with A and the latter with 3′UCU or 3′AUCU (Table 1). 3′UCU was suggested by early experimental studies (Murray and Rabinowitz 1982; Band and Henner 1984), and annotated in the *B. subtilis* genome database SubtiList (http://genolist.pasteur.fr/SubtiList/). However, 3′AUCU appears in *B. subtilis* genomes annotated in GenBank (*e.g.*, NC_000964). A recent study on *B. subtilis* ribosomal structure (*e.g.*, Sohmen *et al.* 2015) also assumed a 3′AUCU tail in ssu rRNA (D. Wilson, personal communication). Existing evidence suggests heterogeneous “mature” ssu rRNA pool given that mature ssu rRNA in bacterial species results from endoribonuclease digestion from the precursor 30S rRNA followed by exonuclease nibbling (Britton *et al.* 2007; Yao *et al.* 2007; Kurata *et al.* 2015). For example, 3′→5′ exoribonucleases such as RNases II, R, and PH, as well as PNPase, all participate in maturation of the 3′TAIL of ssu rRNA (Sulthana and Deutscher 2013), and endoribonuclease YbeY has also been recently shown to participate in the 3′ end maturation of ssu rRNA (Davies *et al.* 2010; Jacob *et al.* 2013). In *E. coli*, 67% of mature ssu rRNA ends with the 3′TAIL in Table 1 (Kurata *et al.* 2015). Thus, the trailing 3′UCU and 3′ACUC may both be present in functional ssu rRNA of *B. subtilis*.

**Table 1:** ssu rRNA 3′ ends that are free to base-pair with SD motifs in *E. coli* and *B. subtilis* and their compatible motifs

Species and 3′ TAIL Sequence*^a^*	SD Motifs*^b^*	
*E. coli*	**U**AAG	
* *3′-**A**UUCCUCCACUAG-5′	**U**AAGG	
	**U**AAGGA	
	**U**AAGGAG	
	**U**AAGGAGG	
	**U**AAGGAGGUG	
*B. subtilis*	**U**AGA	**AGA**A
* *3′-**AUCU**UUCCUCCACUAG-5′	**U**AGAA	**AGA**AA
	**U**AGAAA	**AGA**AAG
	**U**AGAAAG	**AGA**AAGG
	**U**AGAAAGG	**AGA**AAGGA
	**U**AGAAAGGA	**AGA**AAGGAG
	**U**AGAAAGGAG	**AGA**AAGGAGG
	**U**AGAAAGGAGG	**AGA**AAGGAGGU
	**U**AGAAAGGAGGU	**AGA**AAGGAGGUG
	**A**AAG	**GA**AA
	**A**AAGG	**GA**AAG
	**A**AAGGA	**GA**AAGG
	**A**AAGGAG	**GA**AAGGA
	**A**AAGGAGG	**GA**AAGGAG
	**A**AAGGAGGU	**GA**AAGGAGG
	**A**AAGGAGGUG	**GA**AAGGAGGU
	**A**AAGGAGGUGA	**GA**AAGGAGGUG
	**A**AAGGAGGUGAU	**GA**AAGGAGGUGA

a Bolded letters show the differences in the base composition between two species. (*E. coli* ends with A whereas *B. subtilis* ends with UCU or AUCU). The underlined nucleotides denote the alternative 3′-AUCU-5′ TAIL and motifs exclusively compatible with it.

b The SD motifs shown are derived from differences in 3′TAIL (boldface) for both species.

The minor difference in 3′TAIL between *E. coli* and *B. subtilis* suggests different sets of permissible SDs between the two species, *i.e.*, some SDs that function well in one species may not function at all in the other. These species-specific SDs (Table 1) include six in *E. coli* (designated SD_Ec_) and 25 in *B. subtilis* (designated SD_Bs_). Such differences in permissible SDs could contribute to fundamental species differences in translation.

Most *E. coli* mRNAs cannot be efficiently translated in *B. subtilis* (McLaughlin *et al.* 1981a,b), but most *B. subtilis* mRNAs can be efficiently translated in *E. coli* (Stallcup *et al.* 1976). Many gram-negative bacteria, including *E. coli*, can even translate poly(U) messages (Nirenberg and Matthaei 1961; Stallcup *et al.* 1976) but gram-positive bacteria, including *B. subtilis*, cannot translate poly(U) messages (Stallcup *et al.* 1976). In retrospect, it was indeed good luck that Nirenberg and Matthaei (1961) happened to experiment with *E. coli* instead of *B. subtilis*, otherwise the landmark study would have ended up with nothing to report. It is also known that *E. coli* translation machinery can translate leaderless mRNAs (O’Donnell and Janssen 2002; Krishnan *et al.* 2010; Vesper *et al.* 2011; Giliberti *et al.* 2012), and that its 30S ribosomal subunit can still localize the start codon even when the last 30 nucleotides of ssu rRNA is deleted (Melancon *et al.* 1990).

The difference in mRNA permissibility between gram-negative and gram-positive bacteria is often attributed to the presence of the six-domain that is highly conserved RPS1 in gram-negative bacteria (Subramanian 1983), but absent or highly variable in gram-positive bacteria with translation specificity (Roberts and Rabinowitz 1989). RPS1 facilitates translation initiation by reducing secondary structure that could otherwise embed the translation initiation region (TIR) which includes SD and start codon (Roberts and Rabinowitz 1989; Farwell *et al.* 1992; Tzareva *et al.* 1994). *B. subtilis* has a homologous gene with four domains that are not conserved among gram-positive bacteria, with *Mycoplasma pulmonis* and *Spiroplasma kunkelli* having only one domain with weak homology to any known functional RPS1 (Salah *et al.* 2009). These findings corroborate earlier experimental evidence (McLaughlin *et al.* 1981b; Band and Henner 1984) demonstrating that *B. subtilis* requires a more stringent SD region for gene expression than does *E. coli*.

However, the conventional belief that *E. coli* possesses a more permissible translation machinery than *B. subtilis* is not always true. In rare cases, some mRNAs that can be translated efficiently in *B. subtilis* cannot be translated well in *E. coli*, and one such mRNA is gene 6 of the *B. subtilis* phage ϕ29 (Vellanoweth and Rabinowitz 1992). In particular, such translation specificity can often be traced to the 30S ribosome and the mRNAs, rather than other components of the translation machinery, strongly suggesting SD/aSD pairing as the cause for the translation specificity. Indeed, as we show later, gene 6 of phage ϕ29 can form a well-positioned SD/aSD pair only with the 3′TAIL of *B. subtilis* but not with that of *E. coli*. Thus, proper SD/aSD pairing of mRNAs may be the key factor in specifying host specificity of phages, in determining whether a horizontally transferred gene will function in the new genetic background of the host cell, and, ultimately, in speciation and diversification of bacterial lineages.

To facilitate the quantification of optimal positioning of SD/aSD base pairing, we adopted a model of SD/aSD interaction proposed recently (Prabhakaran *et al.* 2015), illustrated with D_toStart_ as a better measure of optimal SD/aSD positioning than the conventional distance between SD and the start codon (Figure 1, A and B). D_toStart_ is constrained within a narrow range in both *E. coli* (Figure 1C) and *B. subtilis* (Figure 1D). This observation serves as a justification for excluding putative SD/aSD matchings lying outside of this range (see *Materials and Methods* section for details).

**Figure 1:**
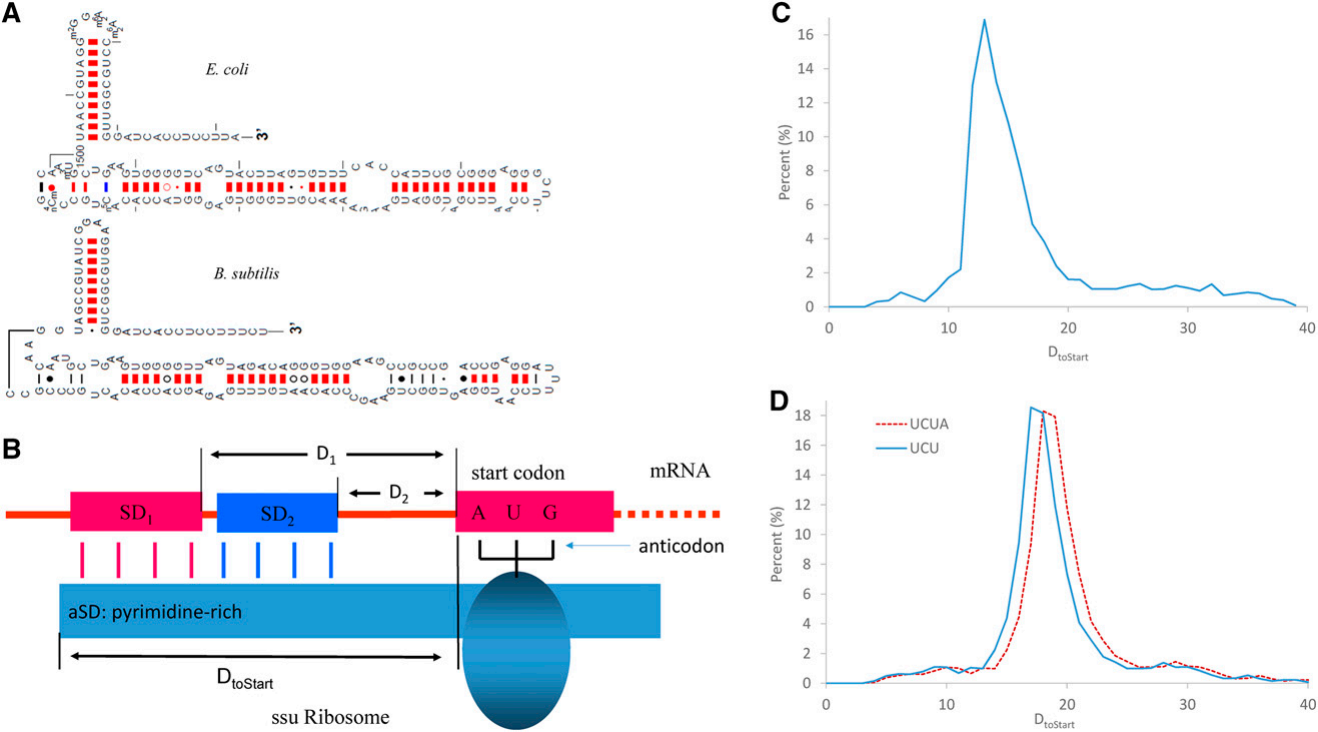
A model of SD sequence and aSD interactions. (A) The free 3′ end of SSU rRNA (3′TAIL) of *E. coli* and *B. subtilis* based on the predicted secondary structure of the 3′ end of the ssu rRNA of *E. coli* and *B. subtilis* from mfold 3.1, adapted from the comparative RNA web site and project (http://www.rna.icmb.utexas.edu). (B) A schematic representation of SD and aSD interaction illustrates D_toStart_ as a better measure for quantifying the optimal positioning of SD and aSD than the conventional distance from putative SD to start codon. SD1 or SD2, as illustrated, are equally good in positioning the start codon AUG against the anticodon of the initiation tRNA, but they differ in their distances to the start codon. D_toStart_ is the same for the two SDs. (C, D) D_toStart_ is constrained to a narrow range in *E. coli* (C) and *B. subtilis* (D); solid blue line denotes SD hits with the UCU-ending TAIL, and the dashed red line shows SD hits with the UCUA-ending TAIL. The *y*-axis in (C) and (D) represents the percentage of SD motif hits detected. See *Materials and Methods* section for details.

The difference in 3′TAIL (Figure 1A and Table 1), and in consequent species-specific compatible motifs (Table 1), between the two bacterial species suggests that selection mediated by 3′TAIL should (1) favor SD_Ec_ in *E. coli* and SD_Bs_ in *B. subtilis*, and (2) be stronger in highly expressed genes (HEGs) than in lowly expressed genes (LEGs). Here, we report results from a comprehensive genomic analysis to test these two predictions.

## Materials and Methods

### Retrieval of genome sequence and protein abundance data

The annotated whole genome sequences for *E. coli K12* (accession number# NC_000913.3) and *B. subtilis 168* (accession # NC_000964.3) in GenBank format were downloaded from the National Center for Biotechnology Information (NCBI) database (http://www.ncbi.nlm.nih.gov). Excluding 180 sequences annotated as pseudogenes in the *E. coli* genome from the analysis resulted in a final total of 4139 genes from *E. coli* and 4175 from *B. subtilis*.

Protein abundance data were retrieved from PaxDB (Wang *et al.* 2012) at www.pax-db.org. The integrated data sets were downloaded for both *B. subtilis* and *E. coli* in order to maximize coverage and consistency scores. We downloaded the paxdb-uniprot-links file relevant to the species (*e.g.*, 224308-paxdb_uniprot.txt for *B. subtilis*), saved the Uniprot ID (the last column) to a file (*e.g.*, BsUniprotID.txt), and browsed to http://www.uniprot.org/uploadlists (last accessed March 7, 2017) to obtain GeneID. Under “Provide your identifiers,” we uploaded the BsUniprotID.txt file, under “Selection options,” we selected the mapping from “UniProtKB AC/ID” to “Gene name” (or GeneID), and clicked “Go”. The STRING identifiers used for each gene in the protein abundance data sets were converted into Gene IDs using UniProt’s retrieve/ID mapping tool (http://www.uniprot.org/uploadlists/) for use in subsequent analyses. The resulting mapping file was generated with two columns (original input Uniprot IDs and the mapped gene name (or GIs GeneID) corresponding to gene name or other IDs in a GenBank file. Unmapped ID is stored in a separate file, also available for downloading.

### HEGs and LEGs

Genes were delimited as HEGs or LEGs on the basis of two metrics: steady state protein abundance levels taken from PaxDB, and I_TE_ (Index of translation elongation) scores computed with DAMBE (Xia 2013) using the default reference files for *E. coli* and *B. subtilis*, which were included in the DAMBE distribution. I_TE_ is advantageous over codon adaptation index (CAI Sharp and Li 1987) or its improved form (Xia 2007b) in that it takes background mutation bias into consideration (Xia 2015). DAMBE’s I_TE_ function has four settings that differ in their treatment of synonymous codon families, and we selected the option breaking sixfold degenerate codon families into four and twofold families. For *E. coli* and *B. subtilis*, the top and bottom 10% of genes for both of these metrics were designated as HEGs and LEGs, respectively.

### Genes of high translation efficiency (HTE) and low translation efficiency (LTE)

HEGs and LEGs defined as above may not be the same as HTE genes and LTE genes. HTE and LTE genes may be characterized by regressing protein abundance on mRNA abundance, so that, given genes with the same mRNA level, those producing many proteins are translated more efficiently than those producing few. The former would be HTE genes, and the latter LTE genes. This requires proteomic and transcriptomic studies carried out with similar bacterial strains, and under similar culture and growth conditions. For *E. coli*, we have used proteomic data from Lu *et al.* (2007) deposited at PaxDB (Wang *et al.* 2012), and transcriptomic data in RPKM (reads per kilobase per million matched reads) from the wild-type strain of *E. coli* (BioProject PRJNA257498, Pobre and Arraiano 2015). For *B. subtilis*, the proteomic data are from Chi *et al.* (2011) deposited in PaxDB and transcriptomic raw counts for three wild-type replicates were downloaded from BioProject PRJNA319983 (GSM2137056 to SM2137058), and then normalized to RPKM. These two transcriptomic studies ignored reads that match to multiple paralogous genes. We have reanalyzed the data with the software ARSDA for analyzing RNA-Seq data (Xia 2017), but the results are nearly identical, partly because there are relatively few paralogous genes in the two bacterial species.

### Identification of anti-SD and SD sequences

The 3′TAILs for *B. subtilis* and *E. coli* used in this paper were based on early empirical evidence (Shine and Dalgarno 1974; Brosius *et al.* 1978; Gold *et al.* 1981; Luhrmann *et al.* 1981; Murray and Rabinowitz 1982; Band and Henner 1984; Tu *et al.* 2009), as well as a series of chemical modification and nuclease digestion experiments that aimed to identify the sequence and secondary structure of bacterial ssu rRNAs using *E. coli* and *Bacillus brevis* (Woese *et al.* 1980). The experimentally derived 3′TAILs for both species are compatible with their corresponding ssu rRNA secondary structure schematics from the Comparative RNA Web Site & Project at www.rna.icmb.utexas.edu, which is curated by the Gutell Lab at the University of Texas at Austin. The schematics include base pairing interactions that are predicted based on the minimum free energy (MFE) state of the structure that in turn were predicted using mfold version 3.1 (http://unafold.rna.albany.edu/?q=mfold; Zuker 2003), with the resulting free 3′ ends shown in Figure 1A.

The sequence of the 3′TAIL used in our analysis for *E. coli* is 3′-AUUCCUCCACUAG-5′ (Shine and Dalgarno 1974; Brosius *et al.* 1978; Gold *et al.* 1981; Luhrmann *et al.* 1981; Band and Henner 1984; Tu *et al.* 2009), because, based on the *E. coli* SSU rRNA secondary structure (Woese *et al.* 1980; Noah *et al.* 2000; Yassin *et al.* 2005; Kitahara *et al.* 2012; Prabhakaran *et al.* 2015), these are the 13 nt at the 3′ end of the ssu rRNA that are free to base pair with the SD sequence. There are two versions of 3′TAIL for *B. subtilis*: 3′-UCUUUCCUCCACUAG (Murray and Rabinowitz 1982; Band and Henner 1984), and 3′-AUCUUUCCUCCACUAG in the genomic annotation. We discussed the possibility of heterogeneous “mature” ssu rRNA pool in the *Introduction*.

### Identification of putative SD sequences

We followed the method of Prabhakaran *et al.* (2015) to identify valid SD sequences, as illustrated in Figure 1. For each gene in each species, we extracted the 30 nt upstream of the star codon and searched matches against the 3′TAIL of the two species by using the “Analyzing 5′UTR” function in DAMBE (Xia 2013). An SD with at least four consecutive nucleotide matches, and positioned with D_toStart_ in the range of 10–22 nt, was considered as a good SD for the *E. coli* translation machinery. For *B. subtilis*, a D_toStart_ range of 12–23 nt was used for the 3′UCU TAIL, or 13–24 nt for the 3′AUCU TAIL. As shown in Figure 1D, the D_toStart_ values for the 3′-AUCU-5′ TAIL in *B. subtilis* are shifted by 1 nt because this measure depends on 3′TAIL length. For this reason, taking 13–24 nt as the optimal range for the 16 nt 3′TAIL is equivalent to using 12–23 nt for the 15 nt 3′TAIL.

### Data availability

All data used to generate the results are available upon request. Software DAMBE for characterizing SD sequences and computing the index of translation elongation (I_TE_), and software ARSDA for characterizing gene expression is available free at http://dambe.bio.uottawa.ca/Include/software.aspx.

## Results and Discussion

*E. coli* has 4323 protein-coding genes (CDSs), with 180 annotated as pseudogenes in the genome and excluded from the analysis, resulting in 4144 functional CDSs. *B. subtilis* has 4175 CDSs with none annotated as pseudogenes. The genomic nucleotide frequencies are 0.2462, 0.2542, 0.2537, and 0.2459, respectively for A, C, G, and T in *E. coli*. The corresponding values in *B. subtilis* are 0.2818, 0.2181, 0.2171, and 0.2830, respectively.

### SD_Ec_ and SD_Bs_ are used more in E. coli and B. subtilis, respectively

As expected, SD_Ec_ are much more frequent in *E. coli* than in *B. subtilis*, with 455 in *E. coli*, in contrast to 267 in *B. subtilis* (Table 2). The difference is highly significant, either against the null hypothesis of equal frequencies (χ^2^ = 48.9529, *P* < 0.0001), against the expected value based on the relative number of CDSs (χ^2^ = 50.3648, *P* < 0.0001; a slightly increased χ^2^ is because *E. coli* has slightly fewer included CDSs than *B. subtilis*), or against the expected values based on both relative number of CDSs and genomic nucleotide frequencies (*e.g.*, AGAA is proportional to P_A_^3^P_G_, AGAAA to P_A_^4^P_G_, and so on, where P_X_ is the genomic frequency of nucleotide X in either *E. coli* or *B. subtilis*), with χ^2^ = 103.07, *P* < 0.0001.

**Table 2:** Number of SD_Ec_ hits (*N*) and their proportion (Prop) in *E. coli* and *B. subtilis* genes

SD_Ec_ motifs	Occurrence in *E. coli*		Occurrence in *B. subtilis*	
	*N*	Prop	*N*	Prop
UAAG	85	0.0205	15	0.0036
UAAGG	91	0.0220	54	0.0129
UAAGGA	151	0.0365	30	0.0072
UAAGGAG	117	0.0283	74	0.0177
UAAGGAGG	10	0.0024	74	0.0177
UAAGGAGGU	0	0	14	0.0033
UAAGGAGGUG	1	0.0002	6	0.0014
Total	455	0.1099	267	0.0640

SD_Ec_, SDs that pair perfectly with the 3′ end of small subunit rRNA from *E. coli*, but not from *B. subtilis*.

The relative abundance of different SDs depends on selection favoring an optimal SD length, and mutations disrupting long SDs. In *E. coli*, the optimal SD length is six (Vimberg *et al.* 2007). *B. subtilis* favors longer SDs. In an experiment with *B. subtilis* with SD lengths of 5, 6, 7, and 12, longer SDs consistently produce more proteins than shorter ones (Band and Henner 1984). This is consistent with the results presented in Table 2, where UAAG is expected to be strongly selected against in *B. subtilis* because it can form only 3 bp against *B. subtilis* 3′TAIL. However, the longer SD_Ec_ is not selected against because an SD_Ec_ such as UAAGGAGG can form 7 bp (except for the first U) against *B. subtilis* 3′TAIL.

Also as expected, SD_Bs_ are also more frequent in *B. subtilis* than in *E. coli*, with 1203 SD_Bs_ in *B. subtilis* in contrast to 576 in *E. coli* (Table 3). The difference is also highly significant (*P* < 0.0001) using the same tests for SD_Ec_ results in Table 2. However, one interesting deviation from the SD_Ec_ data is that SD_Bs_ of length 4 exhibit the opposite pattern, being more frequent in *E. coli* than in *B. subtilis* (Table 3), which assumes a 3′UCU-ending in *B. subtilis* 3′TAIL. The pattern is the same with 3′AUCU-ending of the 3′TAIL (Table S1). This observation can be explained by stronger selection against short SD/aSD in *B. subtilis* than in *E. coli*. Translation efficiency increases with longer and more stringent SD/aSD binding in *B. subtilis*, and such dependence is much stronger in *B. subtilis* than in *E. coli* (Band and Henner 1984). The predicted free energy of SD/aSD for an average *B. subtilis* message is at least 6 kcal/mol more than that of an average SD/aSD in *E. coli* (Hager and Rabinowitz 1985). Thus, a short SD is expected to be selected against, and, consequently, rare in *B. subtilis*, consistent with our results (Table 3), showing that longer SD_Bs_ (5–8 nt) are more frequent in *B. subtilis* than in *E. coli*.

**Table 3:** Number of SD_Bs_ hits (*N*) and their proportion (Prop) in all *Bacillus subtilis* and *Escherichia coli* genes considering UCU as the 3′TAIL

SD_Bs_ motifs	Occurrence in *B. subtilis*		Occurrence in *E.coli*	
	*N*	Prop	*N*	Prop
AGAA	12	0.0029	51	0.0123
AGAAA	66	0.0158	60	0.0145
AGAAAG	60	0.0144	14	0.0034
AGAAAGG	54	0.0129	7	0.0017
AGAAAGGA	60	0.0144	6	0.0014
AGAAAGGAG	28	0.0067	4	0.0010
AGAAAGGAGG	11	0.0026	1	0.0002
AGAAAGGAGGU	1	0.0002	0	0
Subtotal	292	0.0699	143	0.0345
GAAA	16	0.0038	65	0.0157
GAAAG	41	0.0098	28	0.0068
GAAAGG	68	0.0163	18	0.0043
GAAAGGA	51	0.0122	15	0.0036
GAAAGGAG	57	0.0137	10	0.0024
GAAAGGAGG	18	0.0043	1	0.0002
GAAAGGAGGU	3	0.0007	0	0
GAAAGGAGGUG	1	0.0002	0	0
GAAAGGAGGUGA	1	0.0002	0	0
Subtotal	240	0.0575	137	0.0331
AAAG	19	0.0046	38	0.0092
AAAGG	171	0.0410	83	0.0200
AAAGGA	76	0.0182	101	0.0244
AAAGGAG	222	0.0532	64	0.0155
AAAGGAGG	143	0.0343	6	0.0014
AAAGGAGGU	31	0.0074	3	0.0007
AAAGGAGGUG	6	0.0014	0	0
AAAGGAGGUGA	3	0.0007	1	0.0002
Subtotal	671	0.1607	296	0.0715
Total	1203	0.2881	576	0.1391

### Highly expressed genes tend to have longer SDs

**Figure 2:**
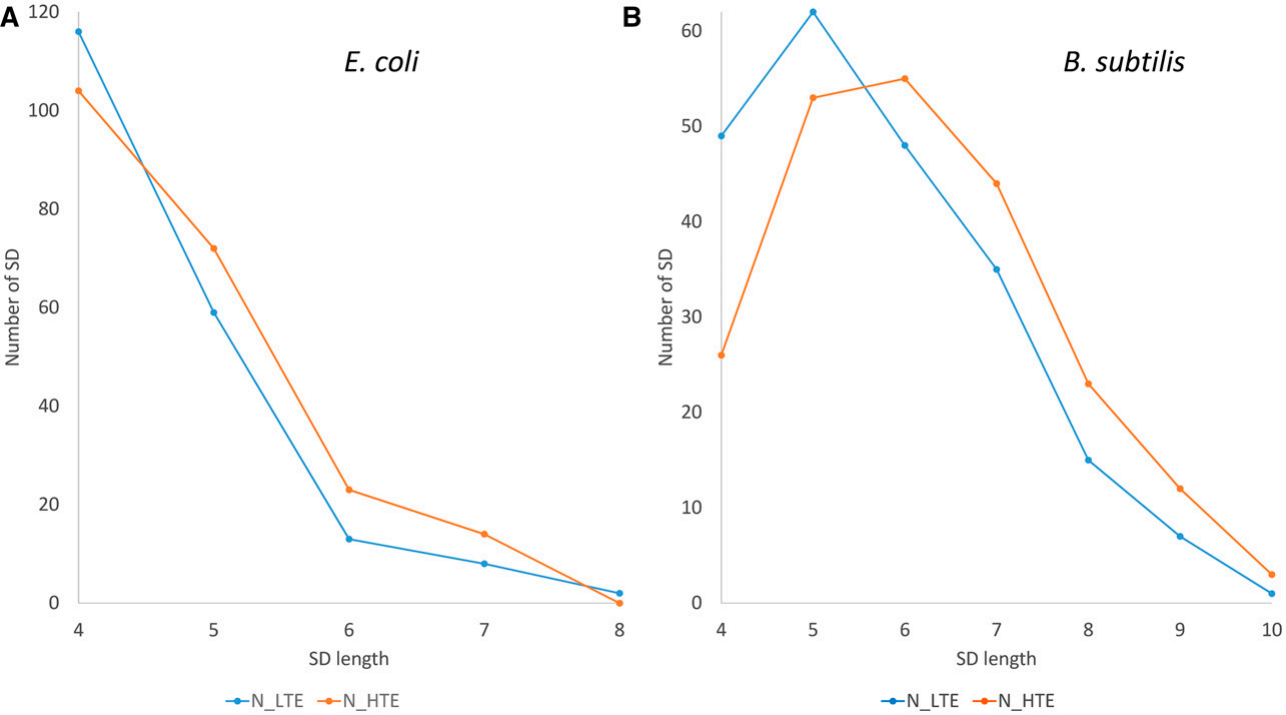
Distribution of SDs from 200 HTE genes and 200 LTE genes over SD length for *E. coli* (A) and *B. subtilis* (B). Classifying genes into HEGs and LEGs generates equivalent results, with HEGs similar to HTE genes, and LEGs similar to LTE genes. HEGs and HTE genes tend to have longer SDs than LEGs and LTE genes.

In addition to the observed difference in SD length between *E. coli* and *B. subtilis* (Figure 2 and Table 3; *B. subtilis* SDs tend to be longer than *E. coli* SDs), there is also clear difference between HEGs and LEGs, or between genes of HTE and of LTE. Although SDs of length four are the most frequent in *E. coli*, longer SDs are relatively more represented in HTE genes than in LTE genes (Figure 2A). This is consistent with previous experimental studies demonstrating an optimal SD length of six (Schurr *et al.* 1993; Komarova *et al.* 2002; Vimberg *et al.* 2007). Optimal SDs in *B. subtilis* are even longer (Band and Henner 1984) than in *E. coli* (Figure 2). We thus expect HEGs or HTE genes to have relatively longer SDs than LEGs or LTE genes, especially in *B. subtilis*. Our empirical results (Figure 2) strongly support this expectation. Short SDs are overrepresented in LEGs and LTE genes, and longer SDs overrepresented in HEGs and HTE genes in both *E. coli* and *B. subtilis*, but more so in *B. subtilis* (Figure 2). This pattern (*i.e.*, association of long SDs with HEGs and HTE genes) is highly significant for *B. subtilis* (chi-square = 12.0375, d.f. = 1, *P*-value = 0.0005214) when tested by the Cochran-Armitage test (Agresti 2002, pp. 181–182) for contingency tables with a linear trend as implemented in the coin package in R (Hothorn *et al.* 2006, 2008). The result for *E. coli*, while consistent with the expectation, is not significant at the 0.05 level (chi-square = 3.3948, d.f. = 1, *P*-value = 0.0654).

### Differential usage of SD_Ec_ and SD_Bs_ in HEGs and LEGs

**Figure 3:**
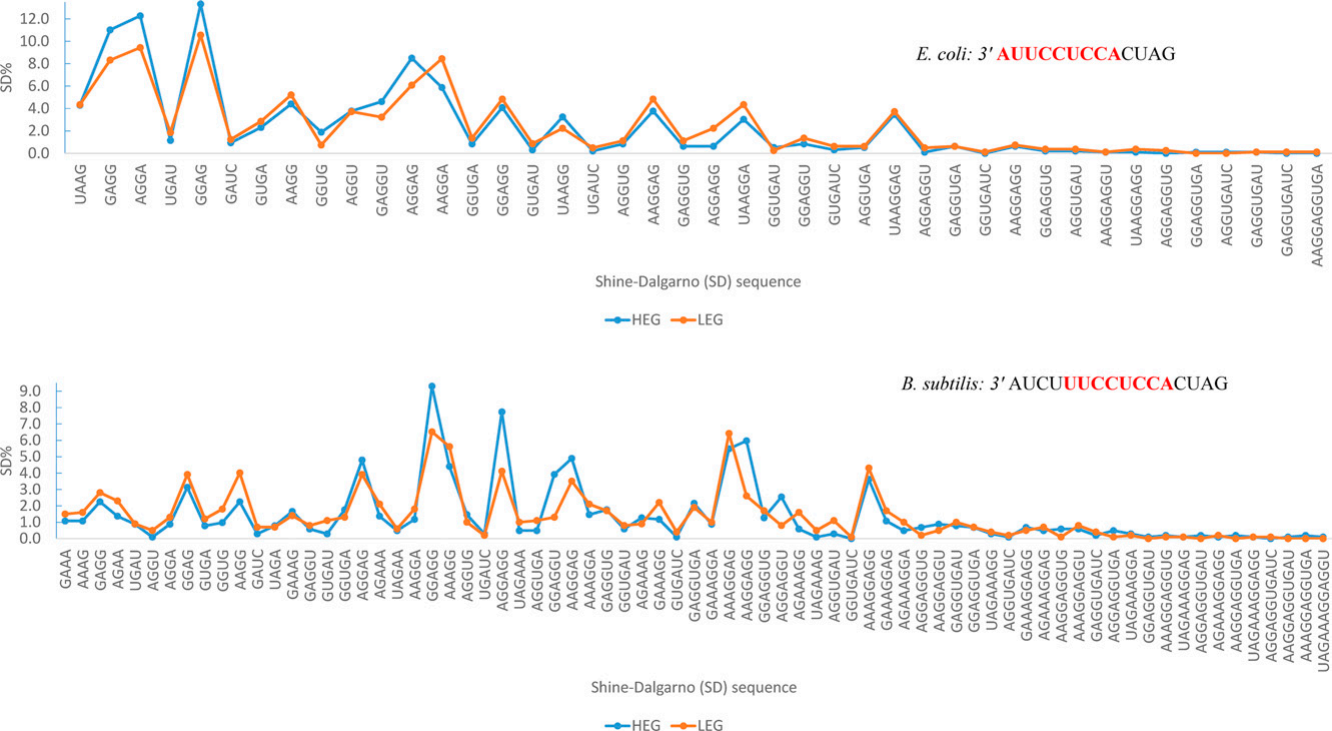
Distribution of *E. coli* and *B. subtilis* SDs for HEGs and LEGs. SDs that are more frequent in HEGs than LEGs match the core aSD (in bold red) of 16S rRNA. The trailing 3′ nucleotides in *B. subtilis* are used mainly for SD/aSD pairing in LEGs. Classifying genes into genes of HTE and LTE generates similar results.

SD_Ec_ is used more frequently in HEGs than LEGs in *E. coli* (Table 4). In contrast, SD_Bs_ is used mainly in LEGs in *B. subtilis* (Table 5), prompting the question of what SDs are used by *B. subtilis* HEGs, and whether the core aSD region (where most HEGs have SD to pair against) for *B. subtilis* HEGs include the trailing 3′UCU (or 3′AUCU). The pattern is similar when contrasting between HTE genes and LTE genes (results not shown). The core aSD region is centered at CCUCC in the overwhelming majority of surveyed prokaryotes (Ma *et al.* 2002; Nakagawa *et al.* 2010; Lim *et al.* 2012). If *B. subtilis* has the same core aSD region, then the trailing 3′UCU (or 3′AUCU) will be used rarely, consequently with few SD_Bs_ pairing to it. The distribution of SDs in *E. coli* and *B. subtilis* is consistent with this interpretation (Figure 3). SDs overrepresented in HEGs relative to LEGs use exclusively 3′AUUCCUCCA as the core aSD region in *E. coli*, and 3′UUCCUCCA as the core aSD region in *B. subtilis* (Figure 3). The trailing 3′UCU (or 3′AUCU) is used as part of aSD mainly by LEGs in *B. subtilis*.

**Table 4:** Number of SD_Ec_ hits (*N*) and their proportion (Prop) in HEGs and LEGs

SD_Ec_ motifs	Occurrence in *E. coli*				Occurrence in *B. subtilis*			
	HEGs		LEGs		HEGs		LEGs	
	*N*	Prop	*N*	Prop	*N*	Prop	*N*	Prop
UAAG	22	0.0053	7	0.0017	1	0.0002	3	0.0007
UAAGG	32	0.0077	6	0.0014	4	0.0010	3	0.0007
UAAGGA	36	0.0087	20	0.0048	3	0.0007	0	0
UAAGGAG	40	0.0097	12	0.0029	9	0.0022	10	0.0024
UAAGGAGG	2	0.0005	1	0.0002	14	0.0034	2	0.0005
UAAGGAGGU	0	0	0	0	0	0	1	0.0002
UAAGGAGGUG	0	0	0	0	4	0.0010	0	0
Total	132	0.0319	46	0.0111	35	0.0084	19	0.0046

**Table 5:** Number of SD_Bs_ hits (*N*) and their proportion (Prop) in highly and lowly expressed genes

SD_Bs_ motifs	Occurrence in *B. subtilis*				Occurrence in *E. coli*			
	HEGs		LEGs		HEGs		LEGs	
	*N*	Prop.	*N*	Prop.	*N*	Prop.	*N*	Prop.
AGAA	0	0	2	0.0005	3	0.0007	3	0.0007
AGAAA	2	0.0005	8	0.0019	7	0.0017	9	0.0022
AGAAAG	6	0.0014	4	0.0010	1	0.0002	1	0.0002
AGAAAGG	3	0.0007	6	0.0014	1	0.0002	0	0
AGAAAGGA	4	0.0010	2	0.0005	2	0.0005	0	0
AGAAAGGAG	2	0.0005	3	0.0007	1	0.0002	0	0
AGAAAGGAGG	1	0.0002	2	0.0005	0	0	0	0
AGAAAGGAGGU	0	0	0	0	0	0	0	0
Subtotal	18	0.0043	27	0.0065	15	0.0036	13	0.0031
GAAA	0	0	2	0.0005	5	0.0012	10	0.0024
GAAAG	2	0.0005	7	0.0017	3	0.0007	1	0.0002
GAAAGG	3	0.0007	11	0.0026	0	0	0	0
GAAAGGA	4	0.0010	5	0.0012	5	0.0012	0	0
GAAAGGAG	2	0.0005	6	0.0014	1	0.0002	1	0.0002
GAAAGGAGG	2	0.0005	2	0.0005	0	0	0	0
GAAAGGAGGU	0	0	0	0	0	0	0	0
GAAAGGAGGUG	0	0	0	0	0	0	0	0
GAAAGGAGGUGA	0	0	0	0	0	0	0	0
Subtotal	13	0.0031	33	0.0074	14	0.0034	12	0.0029
AAAG	1	0.0002	4	0.0010	2	0.0005	2	0.0005
AAAGG	8	0.0019	20	0.0048	7	0.0017	12	0.0029
AAAGGA	5	0.0012	10	0.0024	10	0.0024	9	0.0022
AAAGGAG	17	0.0041	26	0.0062	7	0.0017	7	0.0017
AAAGGAGG	14	0.0033	21	0.0050	1	0.0002	0	0
AAAGGAGGU	2	0.0005	1	0.0002	1	0.0002	0	0
AAAGGAGGUG	1	0.0002	0	0	0	0	0	0
AAAGGAGGUGA	0	0	0	0	0	0	1	0.0002
Subtotal	48	0.0115	82	0.0196	28	0.0068	31	0.0075
Total	79	0.0189	142	0.0335	57	0.0138	56	0.0135

The mature ssu rRNA pool may be heterogeneous in *B. subtilis*. A number of 3′→5′ exoribonucleases, such as RNases II, R, and PH, as well as PNPase, participate in maturation of the 3′TAIL of ssu rRNA (Sulthana and Deutscher 2013), and nuclease YbeY has also been shown recently to participate in the 3′ end maturation of ssu rRNA (Davies *et al.* 2010; Jacob *et al.* 2013). The continuous 3′→5′ digestion implies that the 3′AUCU end will become 3′UCU, 3′CU, and so on. It would make sense for HEGs to use SDs paired with the less volatile part of the 3′TAIL of ssu rRNA (Table 5).

Figure 3, Table 4, and Table 5 suggest that many HEGs in *E. coli* use the species-specific SD_Ec_ and will experience translation initiation problems when translated by the *B. subtilis* translation machinery. In contrast, most HEGs in *B. subtilis* do not use the species-specific SD_Bs_, and will have no translation initiation problems when translated by the *E. coli* translation machinery. Early studies have suggested a more permissible translation machinery in *E. coli* than in *B. subtilis*, *i.e.*, most *E. coli* mRNAs cannot be efficiently translated in *B. subtilis* (McLaughlin *et al.* 1981a,b) but most *B. subtilis* mRNAs can be efficiently translated in *E. coli* (Stallcup *et al.* 1976). The discrepancy in this translation permissibility is often attributed to the presence of the six-domain highly conserved RPS1 in gram-negative bacteria (Subramanian 1983) but absent in gram-positive bacteria with translation specificity (Roberts and Rabinowitz 1989). Our results (Figure 3, Table 4, and Table 5) suggest an alternative explanation for the discrepancy. Because these early studies often involve HEGs, and because *E. coli* HEGs often use species-specific SD_Ec_ (Table 4) whereas *B. subtilis* HEGs rarely use species-specific SD_Bs_, it is not surprising that *E. coli* HEG messages tend to fail in translation initiation in *B. subtilis*, but *B. subtilis* HEG messages tend to have no problem in translation initiation in *E. coli*.

### Species-specific SD and host specificity

One rare exception to the general observation that *E. coli* possesses a more permissible translation machinery than *B. subtilis* is gene 6 (*gp6*) of the *B. subtilis* phage ϕ29, which can be translated efficiently in *B. subtilis* but not *in E. coli* (Vellanoweth and Rabinowitz 1992). Among the 16 nonhypothetical genes in phage ϕ29, *gp6* is the only one that uses a species-specific SD_Bs_ (UAGAAAG) exclusively (Table 6). This SD used all four nucleotides at 3′TAIL of *B. subtilis*, and consequently cannot form SD/aSD in *E. coli* (Table 6). Other genes, such as *gp7* and *gp8*, have two alternative SDs, with one being the species-specific SD_Bs_, but they have another SD that can form SD/aSD binding in *E. coli* (Table 6). Because *gp6* is an essential gene, its use of a SD_Bs_ may explain its host-specificity. That is, even if it gains entry into an *E. coli*-like host, it will not be able to survive and reproduce successfully.

**Table 6:** SD/aSD binding of nonhypothetical genes in *B. subtilis* phage φ29 in *E. coli* and *B. subtilis*

Gene	***E. coli***		***B. subtilis***	
	D_toStart_*^a^*	SD	D_toStart_*^b^*	SD
*gp2*	14	AAGGA	17	AAAGGA
*gp3*	17	AAGGAG	20	GAAAGGAG
*gp4*	18	AGGAGGU	21	AGGAGGU
*gp5*	15	AAGGA	18	AAAGGA
*gp6*			19	UAGAAAG
*gp7*	16	GAGGUGA	18,19	UAGAAAG,GAGGUGA
*gp8*	18	GAGGU	21,21	AGAAA,GAGGU
*gp8.5*	20	GGAGGUG	23	GGAGGUG
*gp9*	16,19	UAAGG,AGGUG	22	AGGUG
*gp10*	15	GAGGUGA	18	GAGGUGA
*gp11*	16	GGUGA	19	GGUGA
*gp12*	15	UAAGGAGG	18	AAGGAGG
*gp13*	17	GAGGU	20	GAGGU
*gp14*	17	AAGGAG	20	AAAGGAG
*gp15*	17	UAAGGAGG	20	AAGGAGG
*gp16*	16	GAGGUG	19	GAGGUG

Gene *gp6*, which uses a species-specific SD_Bs_, cannot form a well-positioned SD/aSD in *E. coli* to be translated efficiently.

a The optimal D_toStart_ is within the range of 10–21 in *E. coli*.

b 3′AUCUUUCCUCCACUAG is used as 3′TAIL for *B. subtilis*, with the optimal D_toStart_ within the range of 15–25.

Another case of host-specificity that may be explained by SD/aSD binding is *E. coli* phage PRD1, which has codon usage deviating greatly from that of its host, in contrast to the overwhelming majority of *E. coli* phages, whose codon usage exhibits high concordance with that of the host (Chithambaram *et al.* 2014). Phage PRD1 belongs to the peculiar Tectiviridae family whose other members, *i.e.*, phages PR3, PR4, PR5, L17, and PR772, parasitize gram-positive bacteria. Phage PRD1 is the only species in the family known to parasitize a variety of gram-negative bacteria, including *Salmonella*, *Pseudomonas*, *Escherichia*, *Proteus*, *Vibrio*, *Acinetobacter*, and *Serratia* species (Bamford *et al.* 1995; Grahn *et al.* 2006). Phage PRD1 is extremely similar to its sister lineages, parasitizing gram-positive bacteria; there is only one amino acid difference in the coat protein between PRDl and PR4 (Bamford *et al.* 1995). It is thus quite likely that the ancestor of phage PRD1 parasitizes gram-positive bacteria. The lineage leading to Phage PRD1 may have switched to gram-negative bacterial hosts only recently, and thus still has codon usage similar to its ancestral gram-positive bacterial host, which is indeed the case (Chithambaram *et al.* 2014). However, one nonhypothetical gene in phage PRD1 (*PRD1_09*) has evolved an *E. coli*-specific SD (UAAG), and does not have alternative SD that can form a well-positioned SD/aSD with *B. subtilis* 3′TAIL. This may have contributed to the host limitation of phage PRD1 within *E. coli*-like species.

The study of coevolution between SD and aSD sequences would be facilitated if 3′TAILs of many bacterial species were characterized experimentally, and if these 3′TAILs differ substantially from each other in different lineages. At present, strong experimental evidence is available for 3′TAIL of *E. coli* and *B. subtilis* (except for the uncertainty on whether the 3′TAIL ends with 3′UCU or 3′AUCU). However, RNA-Seq data may become available for many bacterial species in the near future, and should pave the way for rapid characterization of 3′TAIL of different species by simply mapping the sequence reads to ssu rRNA genes on the genome. One problem to be aware of is that most transcriptomic studies will use an rRNA removal kit to remove the large rRNAs, *i.e.*, 16S and 23S rRNA, in bacteria, because otherwise sequence reads from these large rRNAs will dominate the RNA-seq data. There are two main types of rRNA Remove Kits in the markets: (1) RiboMinus Kit from Invitrogen or MICROBExpress Bacterial mRNA Enrichment Kit (formerly Ambion, now Invitrogen), which have two probes located within the conserved sequence region at each ends of 16S and 23S rRNAs. Full-length rRNA or partial rRNA that pairs with these probes are removed. This implies that such RNA-seq data will lack reads mapped to the 5′ or 3′ ends of ssu rRNAs. The other type of rRNA removal kit is represented by the Ribo-Zero Kit from Epicentre (an Illumina company). This kit removes rRNA across the entire length and does not specifically targets the 5′ and 3′ ends. We used ARSDA (Xia 2017) to confirm that transcriptomic studies using this RNA removal kit have reads that map to the 3′ end of ssu rRNA.

## Supplementary Material

Supplemental material is available online at www.g3journal.org/lookup/suppl/doi:10.1534/g3.117.039305/-/DC1.
