# Supplementary material for: How Changes in Anti-SD Sequences Would Affect SD Sequences in Escherichia coli and Bacillus subtilis
Source: G3 (Bethesda). 2017 Mar 31;7(5):1607–15. doi: 10.1534/g3.117.039305 (PMC5427494; doi:10.1534/g3.117.039305)
Supplement: Supplementary file 1 [file 1607TableS1.pdf]

Table S1. All SD_{Bs} hits with 3'AUCU-ending of the 3'TAIL of *B. subtilis.*

SD_{Bs} motifs	Occurrence in *B. subtilis*		Occurrence in *E.coli*	
	Count	Proportion	Count	Proportion
UAGA	7	0.0017	23	0.0053
UAGAA	13	0.0031	12	0.0028
UAGAAA	22	0.0055	9	0.0021
UAGAAAG	16	0.0038	0	0.0000
UAGAAAGG	13	0.0031	2	0.0005
UAGAAAGGA	17	0.0038	0	0
UAGAAAGGAG	6	0.0014	1	0.0002
UAGAAAGGAGG	2	0.0005	0	0
UAGAAAGGAGGU	1	0.0002	0	0
Subtotal	97	0.0022	47	0.0109
AGAA	10	0.0029	54	0.0125
AGAAA	50	0.0122	77	0.0181
AGAAAG	48	0.0115	20	0.0046
AGAAAGG	42	0.0101	6	0.0014
AGAAAGGA	43	0.0103	6	0.0014
AGAAAGGAG	22	0.0053	3	0.0007
AGAAAGGAGG	9	0.0022	1	0.0002
AGAAAGGAGGU	0	0	0	0
AGAAAGGAGGUG	0	0	0	0
Subtotal	224	0.0544	167	0.0389
GAAA	15	0.0046	74	0.0171
GAAAG	38	0.0091	36	0.0083
GAAAGG	68	0.0163	20	0.0046
GAAAGGA	51	0.0120	16	0.0037
GAAAGGAG	57	0.0137	11	0.0025

GAAAGGAGG	18	0.0043	1	0.0002
GAAAGGAGGU	3	0.0007	0	0
GAAAGGAGGUG	1	0.0002	0	0
GAAAGGAGGUGA	1	0.0002	0	0
Subtotal	252	0.0565	158	0.0366
AAAG	18	0.0060	46	0.0107
AAAGG	162	0.0395	87	0.0201
AAAGGA	76	0.0182	107	0.0248
AAAGGAG	222	0.0532	65	0.0150
AAAGGAGG	143	0.0343	6	0.0014
AAAGGAGGU	31	0.0074	3	0.0007
AAAGGAGGUG	6	0.0014	0	0
AAAGGAGGUGA	3	0.0007	1	0.0002
AAAGGAGGUGAU	0	0	0	0
Subtotal	661	0.1607	315	0.0729
Total	1234	0.2932	687	0.1590
